# Drug-Induced Apoptosis: Mechanism by which Alcohol and Many Other Drugs can Disrupt Brain Development

**DOI:** 10.3390/brainsci3031153

**Published:** 2013-07-31

**Authors:** Catherine E. Creeley, John W. Olney

**Affiliations:** Department of Psychiatry, Washington University School of Medicine, St. Louis, MO 63110, USA; E-Mail: catherinecreeley@hotmail.com

**Keywords:** alcohol, anesthetics, anti-epileptics, apoptosis, fetal alcohol spectrum disorder

## Abstract

Maternal ingestion of alcohol during pregnancy can cause a disability syndrome termed Fetal Alcohol Spectrum Disorder (FASD), which may include craniofacial malformations, structural pathology in the brain, and a variety of long-term neuropsychiatric disturbances. There is compelling evidence that exposure to alcohol during early embryogenesis (4th week of gestation) can cause excessive death of cell populations that are essential for normal development of the face and brain. While this can explain craniofacial malformations and certain structural brain anomalies that sometimes accompany FASD, in many cases these features are absent, and the FASD syndrome manifests primarily as neurobehavioral disorders. It is not clear from the literature how alcohol causes these latter manifestations. In this review we will describe a growing body of evidence documenting that alcohol triggers widespread apoptotic death of neurons and oligodendroglia (OLs) in the developing brain when administered to animals, including non-human primates, during a period equivalent to the human third trimester of gestation. This cell death reaction is associated with brain changes, including overall or regional reductions in brain mass, and long-term neurobehavioral disturbances. We will also review evidence that many drugs used in pediatric and obstetric medicine, including general anesthetics (GAs) and anti-epileptics (AEDs), mimic alcohol in triggering widespread apoptotic death of neurons and OLs in the third trimester-equivalent animal brain, and that human children exposed to GAs during early infancy, or to AEDs during the third trimester of gestation, have a significantly increased incidence of FASD-like neurobehavioral disturbances. These findings provide evidence that exposure of the developing human brain to GAs in early infancy, or to alcohol or AEDs in late gestation, can cause FASD-like neurodevelopmental disability syndromes. We propose that the mechanism by which alcohol, GAs and AEDs produce neurobehavioral deficit syndromes is by triggering apoptotic death and deletion of neurons and OLs (or their precursors) from the developing brain. Therefore, there is a need for research aimed at deciphering mechanisms by which these agents trip the apoptosis trigger, the ultimate goal being to learn how to prevent these agents from causing neurodevelopmental disabilities.

## 1. Introduction

A growing body of evidence generated over the past decade reveals that several classes of drugs frequently used in pediatric and obstetric medicine, including general anesthetics (GAs) and anti-epileptics (AEDs), and drugs that are sometimes abused by pregnant women, including alcohol, PCP, ketamine, barbiturates and benzodiazepines, trigger widespread neuroapoptosis throughout the developing brain of several animal species [1,2,3,4,5,6,7,8,9,10,11], including non-human primates [12,13,14,15,16,17,18,19]. In recent studies we have learned that these same drugs also trigger apoptosis of glial cells, and the vulnerable glial cell type is in the oligodendrocyte (OL) lineage [20,21]. Ols are responsible for myelinating axons that interconnect neurons throughout the brain. The myelin sheath provided by OLs is essential for effective and efficient nerve conduction, and Ols become vulnerable to the apoptogenic action of these drugs at a maturational stage when they are just beginning to engage in myelinogenesis. Apoptotic cell death induced by these drugs in the brains of infant rodents [22,23,24,25,26] or infant monkeys [16] is associated with long-term neurobehavioral disturbances. The non-human primate brain appears to be sensitive to the apoptogenic action of these agents from mid-gestation to a yet-to-be-determined age in infancy or adolescence [12,13,14,15]. These findings raise important questions regarding the role that neuro and oligo apoptosis may play in the fetal alcohol syndrome (FAS), currently referred to as fetal alcohol spectrum disorder (FASD), and also pose a serious question whether other apoptogenic drugs, although long considered safe for pediatric and obstetric use, may have the potential to cause iatrogenic FASD-like developmental disability syndromes. Here we will review evidence addressing both of these questions.

## 2. Fundamental Features of Developmental Drug-Induced Apoptosis

### 2.1. Neuroapoptosis

The neuronal cell death process triggered by these drugs has been demonstrated by various histological cell death markers, including silver, flurojade-B and TUNEL staining, and activated caspase 3 (AC3) immunohistochemistry, and has been shown by electron microscopy to have all the classical morphological characteristics of apoptotic cell death [1,2,3,4,5,6,7,10,11,12,13,14,15,17,18,19,27]. It has been demonstrated quantitatively that neurons are permanently deleted from the developing brain by exposure to these drugs [7,8,26,28], brain volume is permanently reduced [2], and synaptic ultrastructure disrupted [29]. No region of the central nervous system is totally spared, in that the degenerative response has been demonstrated in neurons distributed widely throughout the forebrain, midbrain, cerebellum, brainstem, spinal cord and retina [1,2,4,6,10,30]. The damage is dose- and developmental age-dependent, with several different patterns of degeneration observed, depending on whether drug exposure occurs in early, mid or late synaptogenesis [2,15]. 

Studies using Bax knockout mice and related methods have revealed that drug-induced apoptosis of neurons is Bax-dependent [31] and involves decreased expression of phosphorylated extracellular signal-regulated protein kinase (pERK) [32,33,34], disruption in the dynamic equilibrium between Bax and Bcl_XL _ proteins [34,35], and translocation of Bax to mitochondria causing mitochondrial injury and extra-mitochondrial leakage of cytochrome *c* [31,35]. This is followed by a sequence of changes culminating in activation of caspases 9 and 3 [31,36,37]. In addition to the above evidence implicating the intrinsic mitochondrial pathway, it has also been reported [38] that extrinsic apoptotic pathways may be involved. Results of studies using caspase-3 knockout mice suggest that commitment to cell death occurs before the caspase-3 activation step [37], which signifies that immunohistochemical detection and quantification of neurons positive for activated caspase-3 (AC-3) provides a reliable means of mapping cells that have already progressed beyond the point of cell death commitment. Accordingly, AC-3 immunohistochemistry has become an accepted standard for assessment of dying neurons in recent studies focusing on drug-induced developmental neuroapoptosis [4,7,10,11,12,13,17,26,31,34,36,37,39,40,41,42,43,44,45,46,47].

Several authors have reported evidence that the apoptogenic action of these drugs can be mitigated by pharmacological manipulation of the above-identified intracellular signaling pathways. Dikranian *et al.* [27] presented a detailed ultrastructural description of neurons undergoing apoptotic degeneration following exposure to alcohol, GAs or AEDs, and emphasized that one of the earliest ultrastructural changes was dissolution of the limiting membranes of mitochondria. Subsequently, mitochondria have become a target for developing neuroprotective strategies. For example, Yon *et al.* [35] studied melatonin, a drug that reportedly stabilizes mitochondrial membranes, and found that it reduced the severity of anesthesia-induced neuroapoptosis in infant rats, and Forcelli *et al.* [48] observed a similar protective effect of melatonin against AED-induced disruption of synaptic maturation. Boscolo *et al.* [49] described lytic changes in mitochondrial membranes following anesthesia exposure, and reported that these changes are associated with increased oxidative free radical formation which is damaging to various organelles, including mitochondria. They found that pretreatment with a free radical scavenger or with an agent that restores mitochondrial integrity, prevented these mitochondrial changes and also protected against long-term neurobehavioral consequences of anesthesia exposure. Similarly, Leraci and Herrera [50] have reported that nicotinamide protects against alcohol-induced neurodegeneration in the P7 mouse brain, putatively by a stabilizing action on mitochondrial membranes, and this protective action attenuated long-term neurobehavioral disturbances associated with alcohol exposure. In addition to these protective strategies aimed at preventing mitochondrial injury, others have targeted ERK, an upstream signaling pathway that is known to regulate cell survival. Bittigau *et al.* [3] demonstrated that AEDs suppress ERK phosphorylation in the *in vivo* infant rat brain, suggesting the possibility that preventing pERK suppression might be neuroprotective. Young *et al.* [37] demonstrated that alcohol acutely suppresses pERK in the infant mouse brain, and co-administration of lithium counteracts this pERK suppressant action, while also conferring robust protection against alcohol-induced neuroapoptosis in all regions of the brain. Straiko *et al.* [33] reported that GAs (ketamine and propofol) also suppress pERK, and lithium reverses the pERK suppressant action of these GAs while also preventing them from inducing neuroapoptosis.

A primary goal of neuroprotective strategies is to prevent the apoptosis trigger from being tripped without interfering with the beneficial (anti-convulsant or anesthetic) actions of the apoptogenic drug. The beneficial actions of GAs and AEDs are not fully understood, but it is reasonable to assume that they relate to interactions at the level of the cell surface receptor where the action of the drug suppresses neuronal activity, diminishes transmission of nociceptive and other sensory inputs, reduces motor activity and conscious awareness, *etc*. Action of the drug on these or other receptors also initiates a sequence of complex changes in intracellular signaling pathways, one or more of which serves a cell survival regulatory function. If the changes initiated in cell survival pathways shift the dynamic equilibrium in a direction favoring cell death (apoptosis), there will be a drug-induced pathological increase in the number of cells that die as a result of drug exposure. For a neuroprotective strategy to be successful, it must avoid interfering with drug action at the cell surface receptor level, but be able to intercept and reverse the intracellular apoptosis-prone signal. The strategies described above demonstrate that this is possible by either counteracting the apoptosis-prone signal at the level of mitochondrial injury or at an upstream pERK signaling site. Presumably, interfering upstream is preferable, because this can arrest the apoptosis signal before it triggers mitochondrial injury, whereas interfering at the mitochondrial level runs the risk of being too little or too late to totally avoid mitochondrial injury, cytochrome *c* release and the ensuing apoptosis cascade.

It is noteworthy that while all apoptogenic drugs have in common the property of triggering apoptosis in the developing brain, all such drugs are not equipotent in triggering apoptosis. Thus, one strategy for achieving therapeutic benefits without incurring adverse apoptogenic side effects is to search for drugs that have a wide margin of safety, *i.e.*, a wide separation between the therapeutically effective dose and the dose that causes apoptogenic or other deleterious side effects. It has been reported that some AEDs at clinically relevant doses (*i.e.*, effective anti-convulsant doses) trigger apoptosis and others do not. For example, levetiracetam, at doses that suppress seizures in rats, did not cause a significant apoptosis response in infant rats [51]. However, a recent trial of levetiracetam as a first-line treatment for controlling EEG-confirmed seizures in human neonates raised questions of levetiracetam’s efficacy, in that the majority of infants in the study required that the levetiracetam dosing regimen be supplemented by either one or 2 bolus doses of phenobarbital in order to arrest seizure activity [52]. There is evidence that some anesthetic drugs at equi-anesthetic doses/durations, cause a less severe neuroapoptosis response than others in fetal or neonatal monkeys [12,13,14,45,46,47]. This inspires hope that anesthetic protocols can be developed that provide adequate anesthesia while avoiding, or at least minimizing, apoptosis side effects. However, the volatile halogenated ethers (isoflurane, sevoflurane, desflurane) are the anesthetics of choice for maintaining a sustained deep plane of surgical anesthesia, and in recent studies pertaining to neonatal monkeys [12,13,14,45,46,47], the apoptosis reaction to isoflurane was 3–4 times more severe than the reaction to ketamine or propofol when all three drugs were used at equi-anesthetic doses. Therefore, it remains uncertain for both AEDs and GAs how much improvement in safety can be achieved without compromising therapeutic efficacy. Another complicating factor is that some drug combinations are thought to be more toxic than single drugs (see Section 4.4), so attempting to diminish the toxic action of a potent apoptogenic agent by combining it with a less toxic agent may increase rather than decrease the toxic outcome

### 2.2. Oligoapoptosis

Drug-induced oligoapoptosis has been reported in the fetal monkey brain exposed to alcohol [21] or both the fetal and infant monkey brain exposed to isoflurane, ketamine or propofol [14,20,45,46,47]. Ols undergoing cell death following exposure to alcohol or other apoptogenic drugs are selectively stained immunohistochemically by antibodies to AC3 (marker of apoptosis), or antibodies to fractin (selective for OL apoptosis), DeOlmos cupric silver impregnation (marker of cells that are dead or dying), and can be immunofluorescently double stained with antibodies to AC3 and myelin basic protein (MBP) (marker of Ols which are the only cells in the brain that contain MBP). Ols are the only glial cell type affected; astrocytes and microglia are not vulnerable. All of the above agents trigger oligoapoptosis, and the reaction is confined primarily to white matter regions, but is diffusely distributed throughout the developing brain and spinal cord wherever myelinated axonal pathways are found. OLs in the optic nerve of fetal monkeys have also been found to be vulnerable [20]. It remains to be clarified whether or to what extent mechanisms that underlie the neuroapoptosis phenomenon are also responsible for drug-induced oligoapoptosis.

### 2.3. Windows of Vulnerability

Early research [1,2] focusing on alcohol-induced apoptosis of neurons established that the window of vulnerability for that phenomenon coincided with the brain growth spurt period when billions of recently differentiated neurons are expanding their dendritic surfaces to accommodate incoming synaptic contacts. Thus, vulnerabiliity of neurons appeared to be linked to the period of rapid synaptogenesis, which begins for most neurons in the human brain at some point in the second trimester of gestation. Our present data suggest that vulnerability of OLs is linked to a different functional parameter: myelinogenesis. Therefore, it is possible, indeed likely, that the window of vulnerability for alcohol-induced oligoapoptosis will be found to have a different time schedule—one that corresponds to the progression of myelination events and to the maturational status of OLs that are responsible for the generation and maintenance of myelin. Our present findings indicate that onset of vulnerability to the oligoapoptogenic action of alcohol corresponds not to the time when myelin sheaths are formed, but rather to the earlier time when premyelinating OLs are beginning to generate constituents of myelin, such as MBP, in preparation for myelination of axons [47]. The preparative process in the human brain begins weeks or months prior to onset of myelination in any given brain region [53]. Observations in the fetal NHP brain [47,54] suggest that in some regions, especially in caudal brain regions and in the spinal cord, preparation for myelination begins early in the second trimester. It remains to be determined when the window of vulnerability closes for either the neuro or oligo apoptosis phenomenon, but observations in neonatal monkey brain [12,13,14,20] indicates that it is still wide open for both phenomena one week after birth, which is equal to about 4–6 months after birth for the human brain.

## 3. Relationship between Drug-Induced and Natural Apoptosis

It is well known that apoptosis of neurons and glia is a natural phenomenon during development, which raises the question how drug-induced apoptosis relates to this natural phenomenon. In previous decades, it was believed that failure to make synaptic connections, and/or lack of neurotrophic support, caused up to 50% of neurons to undergo natural cell death during the developmental period [55,56,57]. However, studies using new methods for identifying and counting apoptotic profiles have shown that the bulk of natural cell death occurs in proliferating cell populations that have not yet differentiated into neurons [58,59,60]. Thus, although a high percentage of neural and/or glial precursors may die during development, the vast majority of differentiated neurons become integrated during the period of synaptogenesis, and only a relatively small percentage undergo apoptosis. The original observation that developing neurons are obliged to commit suicide if they fail to make appropriate synaptic connections is accurate, but this observation was made in experiments in which the process of synaptogenesis was intentionally being thwarted experimentally. During normal development, synaptogenesis is not being experimentally thwarted, but exposing developing neurons to alcohol or anesthetic drugs is tantamount to experimental thwarting. It is an unnatural event that can disrupt synaptogenesis or myelinogenesis and cause apoptotic death of many neurons and OLs that would have otherwise survived and made a positive contribution to the functions of the brain. 

An important related question is whether exposure to alcohol or other apoptogenic drugs during early periods of embryogenesis can cause undifferentiated proliferating precursor cell populations to undergo apoptosis at a pathologically increased rate. These cells are already subject to a high rate of natural apoptosis, but is it harmless for unnatural drug exposure to cause these cells to undergo an even higher rate of apoptosis? Sulik and colleagues [61,62] have demonstrated that exposure of *in utero* mouse embyros to alcohol at a gestational age comparable to that of a 3–5 week human embryo does cause a marked increase in apoptotic degeneration of precursor cell populations that are critical contributors to the growth and development of the brain. This issue will be revisited in later see Section 5.5 and Section 7.1. 

## 4. Similarities and Differences between Alcohol and Other Apoptogenic Drugs

### 4.1. Suppression of Neural Activity—A Common Denominator

Most of the apoptogenic drugs identified above either suppress transmission at NMDA glutamate excitatory receptors or promote transmission at GABA_A_ inhibitory receptors, and alcohol has both of these properties. Some of the anti-convulsant drugs do not act directly at these receptors but inhibit excitatory transmission by an action at voltage-gated sodium ion channels [3]. Thus, the most obvious common denominator is that neuroapoptogenic drugs interact either directly or indirectly with the glutamate or GABA transmitter systems in a manner that suppresses neuronal activity. During critical stages of development, neuronal activity is regulated so that neurons can interact in a synchronous manner to meet complex milestones, such as the development of synaptic connections in appropriate timing and sequence. Severe suppression of activity imposed on some neurons, but not others, puts the suppressed neurons out of synchrony with the network they are trying to integrate into, and all neurons are programmed to commit suicide if they fail the task of network integration. OLs in the developing brain also have both glutamate and GABA receptors [63] that may serve to regulate their activity, and we propose that they are similarly programmed to either interact synchronously with neurons to myelinate axons in appropriate timing and sequence, or to commit suicide if they fail to achieve this mission. On a systems integration level this can explain why numerous drugs that suppress neuronal and OL activity all cause a similar adverse reaction in the developing brain—neuronal and OL suicide. 

### 4.2. Competing Hypotheses

There is evidence that in early stages of development, before the NMDA receptor system becomes functionally competent, activation of GABA_A_ receptors may have an excitatory effect [64]. This has given rise to a hypothesis that excess excitation is the mechanism by which GABA agonists trigger neuroapoptosis. Another variation of the excitatory hypothesis has been advanced by Wang and colleagues [19,65,66], who have reported that exposure of cell cultures to ketamine (NMDA antagonist) causes an up regulation of the NR1 subunit of the NMDA receptor. These authors hypothesize that this causes an increase in intracellular calcium leading to cell death by an excitotoxic mechanism. Zorumski and colleagues [67] attempted to evaluate the role of excitation *vs.* inhibition in cell death mediated via GABA_A_ or NMDA receptors, using an *in vitro* postnatal hippocampal neuron culture model. They found that a series of agents, including benzodiazepines and barbiturates, that potentiate GABA-mediated inhibition of neuronal activity, induced cell death which was counteracted by agents that increase excitation or induce increased intracellular calcium levels. In addition, they found that cell death induced by GABA_A_ receptor overactivation was accompanied by a decrease in intracellular calcium levels. They also confirmed a prior finding by Hwang *et al.* [68] that NMDA antagonists trigger apoptotic death of cultured hippocampal neurons which was associated with decreased neuronal activation and decreased intracellular calcium. They concluded that in their *in vitro* model, excessive inhibition mediated through either GABA_A_ or NMDA receptors, and decreased intracellular calcium promote cell death, and this cell death process does not involve increased intracellular calcium or an excitotoxic mechanism. More recently Turner and colleagues [69] have confirmed that neuroapoptosis induced by blockade of NMDA receptors is associated with a decrease in intracellular calcium and is mitigated by pharmacological manipulations that increase intracellular calcium.

Additional observations that argue against the Wang *et al.* hypothesis are that in the *in vivo* rodent brain neurons begin showing robust immunohistochemical evidence for caspase 3 activation (signifying apoptotic cell death) within only several hours after administration of a single dose of ketamine [11], or alcohol [7], whereas evidence reported by Wang *et al.* [19,65,66] indicates that upregulation of the NMDA receptor does not happen until many hours later. In addition, as has been illustrated in many publications, the ultrastructural changes that neurons undergo in response to ketamine, alcohol or other apoptogenic agents, are entirely consistent with apoptotic cell death, and do not resemble the changes that are associated with excitotoxic cell death [1,2,4,6,7,10,27,70]. 

In addition to questions raised regarding the mechanism of cell death, it has been questioned whether the long-term neurobehavioral deficits associated with exposure to apoptogenic drugs are due to apoptosis, or might be due to other accompanying toxic effects of these drugs. For example, it has been reported that administration of alcohol [71] GAs [72] or AEDs [73] to infant rodents causes suppression of neurogenesis which results in a deficit in dentate granule neurons in the adult hippocampus, and long-term memory deficits. These findings have been interpreted as evidence that neurocognitive deficits associated with neonatal exposure to apoptogenic drugs are due, at least in part, to suppression of neurogenesis. A problem with this interpretation is that the doses of drugs used in these experiments are sufficient to cause both neurogenesis suppression and widespread neuroapoptosis throughout many regions of the P7 infant rodent brain. In order to clarify this issue it will require clear-cut, reproducible evidence that neurogenesis suppression and long term neurobehavioral deficits can be produced at doses that do not trigger cell death anywhere in the brain.

### 4.3. Intracellular Signaling Pathways

There have been very few studies comparing alcohol and other apoptogenic agents with regard to the intracellular signaling mechanisms that trigger the apoptosis response in either neurons or OLs. It seems likely that at least some of the pathways and mechanisms underlying the neuroapoptogenic action of alcohol and anesthetic drugs are the same or similar, in view of evidence that alcohol [32], anti-epileptics [3] and anesthetics, including ketamine, propofol and isoflurane [33,34] rapidly suppress ERK phosphorylation in the *in vivo* rodent brain, and lithium prevents this pERK suppressant action, while also preventing both alcohol and anesthetic agents from triggering neuroapoptosis [32,33]. The original studies demonstrating that the apoptotic response is Bax-dependent and is mediated via the intrinsic mitochondrial pathway involving cytochrome *c* release and activation of caspase 3, pertained to neuroapoptosis induced by alcohol, but it has been well documented in subsequent studies that apoptosis induced by anesthetic drugs is also mediated by Bax protein [66] acting via the intrinsic mitochondrial pathway [74] and entails activation of caspase 3 [11,12,13,14,17,18,19,20]. 

### 4.4. Toxic Synergism of Drug Combinations

An interesting feature of drug-induced neuroapoptosis is that the apoptotic reaction induced by a given apoptogenic drug can be substantially augmented by combined administration of that drug with certain other drugs. A prime example of this phenomenon is observed when caffeine is administered together with alcohol or various anesthetic or anticonvulsant agents. The mechanism underlying this effect is poorly understood. Caffeine, by itself, at relatively high doses has been shown to trigger apoptosis [75,76], but at lower doses that show little or no apoptogenicity, it markedly potentiates the apoptogenic action of alcohol or various GAs [77,78]. Another peculiar example of potentiated apoptogenicity is observed when morphine and caffeine are combined. Morphine by itself is not apoptogenic, but if morphine is administered with an apoptogenic dose of caffeine, the apoptogenicity of caffeine is potentiated [76]. A related phenomenon is seen when certain GAs are combined with one another. For example, nitrous oxide (laughing gas), at clinically relevant doses, has not been shown to induce neuroapoptosis, but when nitrous oxide is combined with isoflurane it potentiates the apoptogenicity of isoflurane [6,18,39]. Similarly, the AED, levetiracetam, at a low clinically relevant dose does not trigger neuroapoptosis in the infant rat brain, but when this dose of levetiracetam is combined with a therapeutic dose of phenobarbital or phenytoin, it augments their neurotoxic response [79].

These toxic synergism findings have potentially important implications in a public health context because it is common practice in neonatal intensive care units (NICUs) worldwide to expose premature infants intermittently or continuously to anesthetic and analgesic drugs (procedural sedation), while also exposing these infants semi-chronically (days or weeks) to high doses of caffeine in order to stimulate respiration and prevent apneic spells, to which these infants are prone (apnea of prematurity). When these infants require a surgical procedure it is common practice to use an anesthetic cocktail consisting of several GAs, such as isoflurane + nitrous oxide + midazolam, a combination that has been shown in rodents to cause a severe apoptotic reaction [6,18,34]. In addition, some of these infants manifest epileptic seizure activity for which they are often treated with a cocktail of AEDs, and because AEDs depress respiration, they may be treated with caffeine to counteract respiratory depression. It is well documented that children with a history of premature birth and prolonged care in the NICU, have a high incidence of neurocognitive impairment [80], but drugs used in the NICU for procedural sedation, surgical anesthesia, control of seizure activity, or to stimulate respiration have, thus far, escaped scrutiny as potential contributory factors. Therefore, there is an urgent need for research aimed at clarifying mechanisms underlying synergistic interactions of neuroactive drugs used in the NICU. The observation that caffeine markedly potentiates the neurotoxicity of alcohol is of potential concern in a public health context in that caffeine and alcohol are the two most commonly used and abused drugs in modern society, and it is not uncommon for pregnant mothers to expose their fetuses to coffee or other sources of caffeine while they are also ingesting alcoholic beverages.

It has been postulated that there may be an interactive relationship between the pain of surgery and the toxic action of anesthetic drugs. There are two opposing schools on this issue. Anand and Soriano [81] proposed that anesthesia unaccompanied by surgical pain or any painful stimulus may result in a more severe neuroapoptosis reaction than anesthesia accompanied by surgery or a painful stimulus. Shu *et al.*, [82] recently tested this hypothesis and were not able to confirm it. In fact, they found the exact opposite—that anesthesia in the absence of surgical pain or any other source of pain caused less neuroapoptosis than anesthesia accompanied by surgery or accompanied by a persistent painful stimulus.

### 4.5. Relating Patterns of Cell Loss to Long-Term Neurobehavioral Disturbances

**Figure 1:**
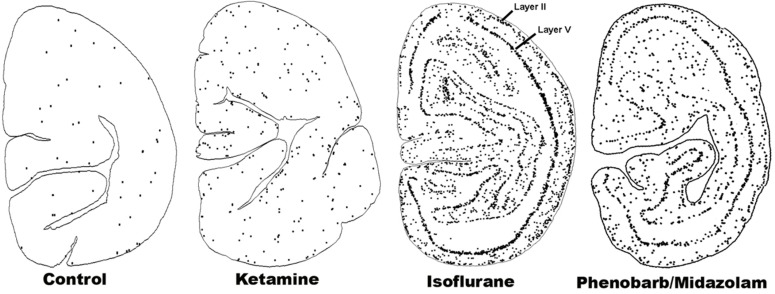
Computer plots showing laminar degeneration of layer II and V neurons in the primary visual cortex of neonatal rhesus monkeys following exposure to GABA agonists, and absence of a similar pattern in control or ketamine-exposed neonatal monkey brain.

**Figure 2:**
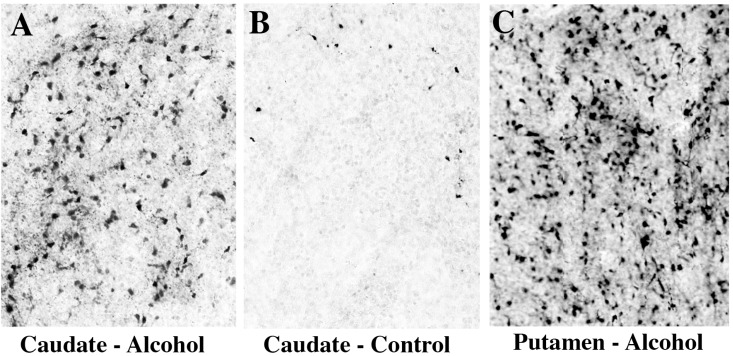
Cellular degeneration induced by alcohol in the caudate/putamen of fetal rhesus monkey brain as detected by activated caspase 3 (AC3) immunohistochemistry. There is a marked increase in AC3-positive neuronal profiles in the alcohol-exposed caudate (**A**) and putamen (**C**), compared to the sparse display of apoptotic profiles in control caudate (**B**) or control putamen (not shown).

**Figure 3:**
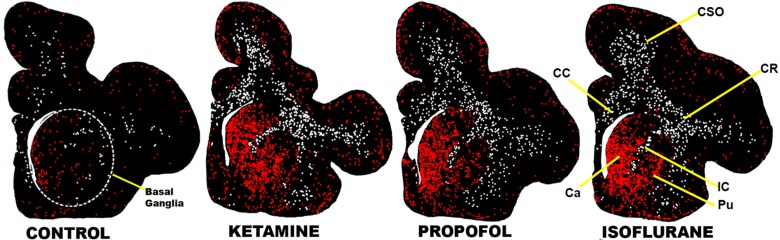
These panels are computer plots of sections cut through the basal ganglia [caudate (Ca) and putamen (Pu)] of the fetal rhesus monkey brain following exposure to an NMDA antagonist anesthetic (ketamine), or to GABA agonist anesthetics (propofol or isoflurane). Apoptotic neurons (red dots) are sparsely distributed in the basal ganglia region of the control brain, and are much more heavily concentrated in the basal ganglia region following exposure to any of the three anesthetic agents. In the anesthesia-exposed brains, apoptotic OLs (white dots) are densely and diffusely distributed throughout all white matter areas, including the corpus callosum (CC), Corona Radiata (CR), Centrum Semi-Ovale (CSO) and Internal capsule (IC).

Some neurons have NMDA receptors and do not have GABA_A_ receptors or vice versa, and some have both, which provides a basis for different cell populations being affected by these two classes of drugs, and also for overlap in the cell populations affected. There is evidence that alcohol [2,7], which interacts with both NMDA and GABA receptors, or an anesthetic cocktail that contains both an NMDA antagonist and a GABA agonist [6,23] tend to produce particularly severe cell loss reflecting simultaneous interaction with both classes of receptors, either on the same or on different cells. In early rodent studies it was demonstrated that an NMDA antagonist and GABA agonist induce neuroapoptosis in the cerebral cortex and thalamus, but each agent induced its own pattern of cell loss within each of these brain regions, and alcohol induced a pattern comprising a composite of the two patterns [2]. More recently we have observed a striking example of receptor-specific patterns of neuroapoptosis in the neonatal NHP brain [12,13,14,45,46], with a pattern of dense laminar degeneration of layer II and V neurons in the primary visual cortex following exposure of neonatal rhesus monkeys to isoflurane or a combination of phenobarbital and midazolam (all of which are primarily GABA agonists), whereas exposure to ketamine (NMDA antagonist) had very little effect on these neuronal populations (see Figure 1). In the fetal NHP brain there are several neuronal populations that are severely affected by both NMDA antagonists and GABA agonists and by alcohol. The most notable examples are the basal ganglia (see Figure 2 and Figure 3) and cerebellum which show severe loss of the same neuronal populations following exposure to any of these agents [13,15,47,54].

## 5. Can Alcohol Apoptogenicity Explain Signs and Symptoms of FASD?

It is well recognized that *in utero* exposure of the human fetus to alcohol can cause a syndrome of deleterious effects currently referred to by the acronym FASD. The full syndrome includes craniofacial malformations, structural pathology in the brain and long-term neurobehavioral disturbances. The behavioral disturbances manifest in childhood as attention deficit/hyperactivity disorder (AD/HD) and learning impairment, ranging in severity from mild to very extreme [83,84]. In addition, individuals diagnosed with FASD have a high incidence and wide variety of adult-onset psychiatric disorders, including a 40% incidence of psychosis and 44% incidence of major depressive disorder [85]. Given the abundance of recent evidence that alcohol triggers widespread apoptotic cell death of fully differentiated neurons and OLs in the developing animal brain, including the fetal primate brain, the question arises whether, or to what extent, the apoptogenic properties of alcohol are responsible for the signs and symptoms of FASD. Lines of evidence suggesting that apoptosis plays a major contributory role are as follows. 

### 5.1. Reduction in Brain Mass

**Figure 4:**
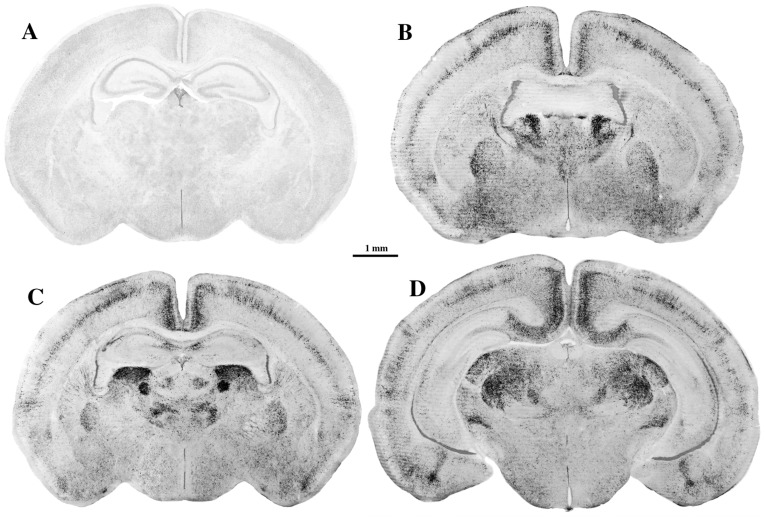
Coronal sections of neonatal mouse brain 24 h after exposure to saline (**A**) or alcohol (**B**–**D**). All sections stained by DeOlmos cupric silver method (marker of dead or dying cells). Sections **B**–**D** are cut at 3 rostro-caudal levels to show the massive extent and remarkable bilateral symmetry of alcohol-induced neurodegeneration.

Both autopsy and neuroimaging studies have consistently described a reduction in brain mass as a finding that typifies the FAS syndrome [84,86]. In 1990, Goodlett *et al.* [87] exposed infant rats on a single day during the brain growth spurt period (third trimester equivalent) to a high dose of alcohol and observed that it caused a significant “restriction” in brain weight and a loss of cerebellar Purkinje cells. One decade later, Ikonomidou *et al.* [2] administered a similarly high dose of alcohol to infant rats and confirmed that it caused a significant reduction in brain mass, but they also examined the brain in the acute post-treatment interval and found evidence for acute apoptotic degeneration of neurons, not only in the cerebellum, but on a massive scale throughout many other regions of the developing brain (see Figure 4). Widespread apoptotic cell death throughout many regions of the brain following a single exposure to alcohol has now been confirmed in third trimester fetal monkeys [15]. It stands to reason that if a single exposure to alcohol during the third trimester equivalent can cause sufficient cell loss to significantly reduce the mass of the brain, multiple exposures to alcohol, as commonly occurs in fetuses of mothers who have a strong alcohol habit, can cause even a greater loss of cells and reduction in brain mass. These findings leave little doubt that one cardinal feature of FASD neuropathology—reduction in brain mass—can readily be explained, at least in part, by exposure to alcohol during the third trimester of gestation.

### 5.2. Focal Impact on Basal Ganglia

**Figure 5:**
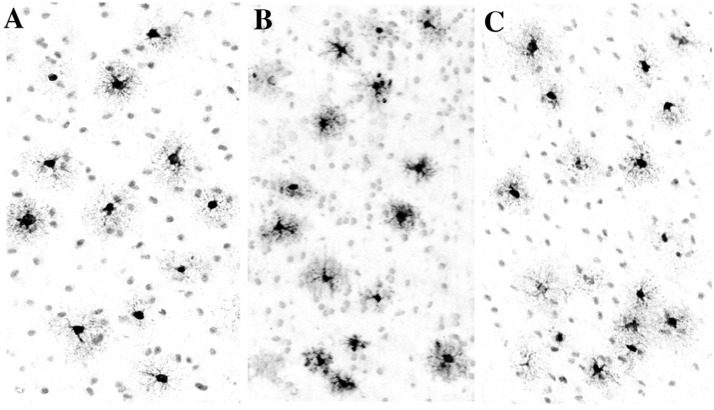
Sections from the internal capsule (**A**); corpus callosum (**B**); and corona radiata (**C**) of a G120 fetal macaque 8 h after exposure to alcohol, showing that in each of these white matter regions there are abundant oligodendroglia (OLs) that stain positive for activated caspase 3 (AC3), signifying that they are dying by apoptosis. Within 5 h after alcohol is administered, large numbers of OLs in diffusely scattered distribution throughout the white matter become AC3 positive, then rapidly progress to an advanced stage of degeneration in which they become fragmented and reduced to particulate debris that is rapidly phagocytized by macrophages and removed from the scene.

Neuroimaging studies [84,86] have consistently identified the basal ganglia (caudate/putamen) as a brain region that shows a particularly prominent loss of neuronal mass in FASD patients. In infant rodents [2,7,36], at a brain age equivalent to a third trimester human fetus, and in third trimester fetal monkeys [15], a single exposure to alcohol causes an apoptotic neurodegenerative reaction in the caudate nucleus and putamen that is typically more severe than in other regions of the brain (see Figure 2 above). If a single exposure of the third trimester fetal monkey brain to alcohol causes a very severe apoptotic cell death reaction focally impinging upon basal ganglia neurons, multiple exposures during this period would cause the loss of neuronal mass in this region to be more extreme, and would increase the chances that the focal impact of alcohol on this brain region would be detectable by subsequent neuroimaging evaluation. An additional relevant factor is that the caudate and putamen, which are major contributors to the basal ganglia mass, are separated by a large corridor of white matter (the internal capsule) that also sustains a loss of mass due to alcohol-induced apoptotic deletion of OLs from this structure (see Figure 5A). This would increase the overall appearance of mass reduction in the basal ganglia region. Consistent with this observation, in a neuroimaging study pertaining to FASD brain changes, Archibald and colleagues [86] described a disproportionately large reduction in mass of the basal ganglia, and also made the novel observation that, in general, loss of cerebral volume was greater in white matter than gray matter regions. These several findings support the interpretation that alcohol has a predilection for deleting both neurons and OLs from the basal ganglia region of the fetal primate brain during the third trimester of gestation, and this can account for at least some of the decreased basal ganglia mass that has been reported as a prominent feature of FASD brain changes.

### 5.3. Impact on Corpus Callosum

**Figure 6:**
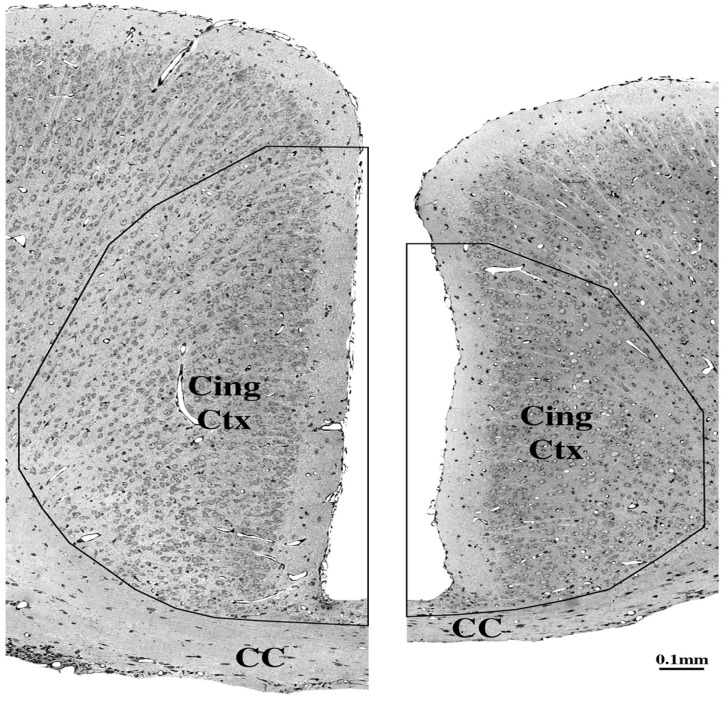
These panels depict the cingulate cortex and corpus callosum of a 10-day-old mouse brain, 72 h after treatment with saline (left) or a single high dose (5 g/kg) of alcohol (right). Both brain sections are shown at the same magnification and are cut from the same rostrocaudal level. Note the decreased cortical mass and also the decreased size of the corpus callosum of the alcohol brain. The number of neuronal profiles within the demarcated area of the saline *vs.* ethanol brain is 881 *vs.* 488, which represents a 45% cell loss within the cingulate region. Adapted from Olney *et al.* [7].

**Figure 7:**
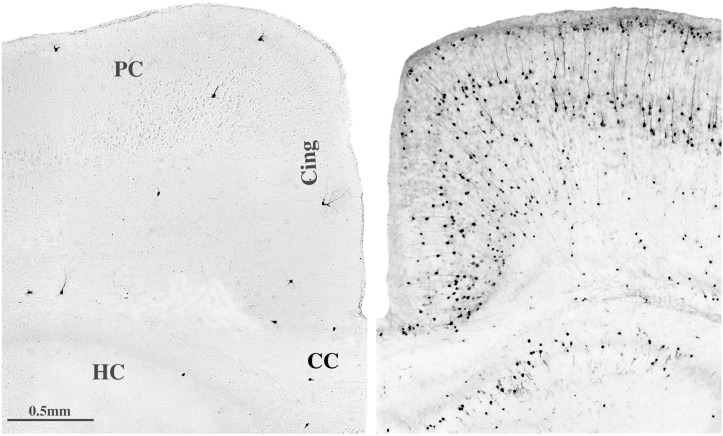
These histological sections depict the parietal cortex (PC), cingulate cortex (Cing), rostral hippocampus (HC) and corpus callosum (CC) of a 7-day-old C57BL/6 mouse 8 h following subcutaneous treatment with saline (left) or ethanol (right). Both sections have been stained immunocytochemically with antibodies to activated caspase-3. The saline control brain shows a pattern of caspase-3 activation that occurs normally in the 7-day-old mouse brain, and is attributable to physiological cell death. The pattern of caspase-3 activation closely resembles the pattern of silver staining shown in Figure 4C, but the density of caspase staining is not as great as the density of silver staining, because the silver stain marks all neurons and fragments thereof that have degenerated over a 24 h period, and caspase-3 marks only those neurons that are undergoing caspase-3 activation at a given survival interval, in this case the 8 h interval. Adapted from Olney *et al.* [7].

Agenesis or dysgenesis of the corpus callosum has been reported as an anomaly sometimes observed in severe cases of FAS. While gross structural anomalies are usually believed to arise from a disruptive influence during early embryogenesis, Livy and Elberger [88] exposed rats *in utero* to daily doses of ethanol throughout gestation (equivalent to the first and second trimesters of human gestation) and found that it had no effect on the dimensions of the corpus callosum, although it did reduce the size of a related structure, the hippocampal commissure. Archibald *et al.* [86] reported neuroimaging evidence that both the parietal cortex and the corpus callosum are reduced in size in FASD patients, and we have been able to reproduce this finding by administering a single dose of alcohol to infant mice when they are at an age equivalent to a third trimester human fetus. Our evidence documents that in addition to a marked reduction in the caliber of the corpus callosum (see Figure 6), large numbers of neurons from the middle layers of the parietal cortex are deleted bilaterally (see Figure 7). These neurons communicate reciprocally from one hemisphere to the other via the corpus callosum, and their axons constitute a significant percentage of the fibers that comprise this structure. Deletion of such neurons will understandably diminish the size of the corpus callosum, and if exposure to alcohol occurs more than once during the vulnerable period, presumably each exposure will delete additional axons from the corpus callosum. More recently we have demonstrated [21] in the third trimester fetal monkey brain that a single dose of alcohol triggers apoptotic degeneration of large numbers of OLs in the corpus callosum, and in the centrum semi-ovale and corona radiata (see Figure 5B,C above), which are large WM pathways that feed into the corpus callosum. We found these changes to be particularly severe in the subcortical white matter of the parietal lobe, which is consistent with the observation of Archibald *et al.* [86] that reductions in white matter volume are particularly pronounced in the parietal lobe of FASD subjects. Destruction of OLs in the parietal white matter of both hemispheres at a time when they are just beginning to myelinate axons that are destined to course through the corpus callosum, provides an additional reason for believing that the apoptogenic action of alcohol during the third trimester can cause dysgenesis and a reduced mass of the corpus callosum. 

### 5.4. Long-Term Neurobehavioral Disturbances

Children diagnosed with FASD typically manifest a heterogeneous variety of neurobehavioral disturbances, including symptoms relevant to AD/HD and/or learning disability [84,85], and in adulthood, FASD is associated with a high incidence of psychosis and depressive disorder [85]. As indicated above, there is evolving evidence that exposure of infant rodents during the third trimester equivalent to NMDA antagonist drugs causes long-term disturbances primarily in behavioral domains relevant to AD/HD [22,77], whereas exposure to GABA agonists causes long-term disturbances primarily in behavioral domains relevant to learning disability [78]. Evidence that alcohol causes patterns of apoptotic cell loss in the developing rodent brain that incorporate features of both an NMDA antagonist pattern and a GABA agonist pattern [2], suggests an explanation for the observation that FASD children frequently manifest neurobehavioral disturbances in both AD/HD and learning disability domains. The observation that FASD subjects have a high incidence of adult-onset psychiatric disorders is also understandable in terms of the third trimester proapoptotic action of alcohol. Psychological, emotional and mental behaviors are the functional expression of neurons and neural networks interacting with one another. It is logical to expect that destruction of neurons by alcohol during a developmental stage when they are in the process of integrating into neural networks, will derange the organization of these networks, thereby setting the stage for subsequent dysfunctional behaviors. 

The developing brain has plasticity properties that may allow it to compensate for neuronal losses if the losses are not too severe. Chances for recovery of function may be especially favorable following unilateral focal neuronal losses, because homologous neuronal groups from the contralateral hemisphere may support functional recovery. However, the pattern of damage induced by alcohol (see Figure 4), and other drugs that mimic alcohol’s apoptogenicity, features bilaterally symmetrical neuronal losses from both hemispheres. The same neuronal groups that are decimated on one side of the brain are decimated on the other side as well, and this may severely limit the capacity for functional recovery. This feature of alcohol’s neurotoxic action increases the probability that alcohol-induced neuronal losses will be reflected in permanent long-term neurobehavioral disturbances. It is understandable that the dysfunctional behaviors associated with fetal alcohol exposure will be heterogeneous and difficult to fit into specific diagnostic categories because no two individuals with FASD will have the same profile of genetic predispositions or the same history regarding the timing, number and magnitude of alcohol exposures.

### 5.5. Craniofacial Dysmorphism

Differentiated neurons and OLs become vulnerable to alcohol’s apoptogenic action approximately in mid gestation, and they remain vulnerable throughout the remainder of gestation. Therefore, the evidence reviewed above, supports the interpretation that alcohol, by exerting an apoptogenic action on differentiated neurons and OLs during the second half of gestation can account for many of the pathological manifestations of FASD. However, a major feature that cannot be explained by the apoptogenic action of alcohol impinging upon the *in utero* fetus during the second half of gestation, is craniofacial malformations and related dysmorphogenic anomalies that occur in some FAS individuals. 

In a series of studies, Sulik and colleagues [61,62] have presented compelling evidence that FAS-like craniofacial malformations can be reproduced in the mouse embryo by *in utero* exposure to alcohol during the 7th to 9th days of gestation, which corresponds to the 3rd to 4th weeks of human gestation. In addition, they show that these craniofacial dysmorphic features are accompanied by brain changes that correlate with structural anomalies that have been reported in FASD. Does this contradict our proposal that the apoptogenic properties of alcohol can explain many of the signs and symptoms of FASD? No, it lends substantial support to this interpretation because evidence presented by Sulik *et al.* [62] identifies apoptosis as the mode of cell death induced by alcohol during early embryogenesis. Their findings are consistent with the interpretation that during the proliferative stages of development: when these precursor cells are subject to a high rate of natural apoptosis, alcohol disrupts their progress in meeting critical milestones and, thereby, shifts the rate of apoptosis from a high natural rate to a much higher pathological rate. Since these cells are progenitors that give rise to future generations of neurons and glia, deleting them in this early proliferative stage would be expected to deprive the brain of functional components needed in later stages for the formation of normal neural networks. 

We propose that the bulk of evidence is consistent with the interpretation that the structural and functional organization of many different brain regions can be disrupted by alcohol exposure at any of several stages in the evolution of a given brain region, and the resulting derangement will affect behavioral outcome to a relatively subtle or extremely unsubtle degree, depending on many factors, including the developmental age of the conceptus at the time of exposure, the number of exposures, the magnitude and duration of blood alcohol elevations, and on genetic factors that can increase or decrease susceptibility to the disruptive influence of alcohol.

## 6. Can Other Apoptogenic Drugs Cause FASD-Like Syndromes?

A growing body of evidence cited above from animal studies suggests that anesthetic and anticonvulsant drugs mimic alcohol in causing neuro and oligo apoptosis if exposure occurs during developmental stages corresponding in humans to a period from mid gestation to several years after birth. Millions of human fetuses and infants are exposed every year worldwide to these drugs during this developmental period. This raises the important question whether these drugs could be causing FASD-like syndromes without our being aware of it. The following information pertaining to anesthetic and anti-convulsant drugs will help the reader evaluate this question:

### 6.1. Anti-Epileptic Drugs (AEDs)

#### 6.1.1. Late Gestation Structural Brain Changes, Including Focal Impact on Basal Ganglia

Most AEDs are either GABA agonists or blockers of voltage-gated sodium ion channels. Both categories trigger an apoptosis response in the developing rodent brain [2,3], and this is also true for those that have been tested, primarily GABA agonists, in the developing monkey brain [89]. AEDs mimic alcohol in having a predilection for deleting large numbers of neurons from the basal ganglia of both infant rodents [2,3,11] and fetal monkeys [89], which correlates with the fact that reduced basal ganglia mass is one of the most consistent findings in neuroimaging studies of FASD subjects [84,86]. Ikonomidou and colleagues [90] have demonstrated by neuroimaging methods that *in utero* exposure of human fetuses to AEDs in the third trimester of gestation is associated with a significant loss of neuronal mass in the basal ganglia. Thus, there is relatively strong evidence that third trimester AED exposure reproduces in both animals and humans the same structural brain pathology, including focal reduction in basal ganglia mass, that is often described in association with FASD.

#### 6.1.2. Early Gestation Teratogenic Effects

Treatment of mothers who have epilepsy with AEDs, especially valproate, in the first trimester of gestation, is associated with a wide range of teratogenic effects, including cleft lip and palate and neural tube defects, but a full FAS-like craniofacial dysmorphism syndrome has not been described. However, first trimester AED exposure occurs only at low doses (to avoid over-sedation of the mother) in contrast to alcohol exposure, which may include exposure to high blood alcohol levels for many hours, sometimes on multiple occasions. Therefore, it remains unclear whether AEDs have the potential to reproduce FAS-like teratogenic effects during the first 4 weeks of human gestation.

#### 6.1.3. Neurobehavioral Disturbances

There is longstanding evidence [91,92] that daily treatment of young children with phenobarbital at a relatively low dose (4–5 mg/kg/day) is associated with a small but significant deficit in IQ. More recent evidence from a multicenter study, documents that daily exposure of third trimester human fetuses to valproate, which is one of the most potent AEDs in triggering neuroapoptosis in the developing rodent brain [3], is associated with a 9 point deficit in IQ [93,94]. In that same study, it was found [94] that third trimester exposure to lamotrigine was associated with long-term impairment in verbal abilities, which is consistent with findings of Forcelli *et al.* [48,95] that lamotrigine exposure of infant rats disrupts synaptic maturation and is associated with long-term neurobehavioral disturbances. Thus, exposure of young children or third trimester human fetuses to AEDs can cause FASD-like long-term neurocognitive deficits. 

### 6.2. Gerneral Anesthetics (GAs)

#### 6.2.1. Late Gestation Structural Brain Changes, Including Focal Impact on Basal Ganglia

All GAs have either NMDA antagonist or GABA agonist properties, and exposure to any of these agents during the brain growth spurt period mimics alcohol in triggering both neuroapoptosis and oligoapoptosis in the developing rodent and monkey brain, with long-term adverse neurobehavioral consequences. GAs also mimic alcohol in preferentially deleting large numbers of neurons from the basal ganglia region of the infant rodent [6,11,38,41] or fetal monkey brain [13,47,54] (see Figure 4), and thereby reproduce in animal brain an important structural change that frequently has been described in the brains of FASD subjects.

#### 6.2.2. Early Gestation Teratogenic Effects

The literature suggests that anesthesia in early pregnancy does not pose significant risk of causing teratogenic effects, but the exposure conditions evaluated in the reported studies were not comparable to alcohol exposure conditions. A mother who is given to binge drinking may expose her fetus early in pregnancy to very high blood alcohol levels that are sustained for many hours, whereas the literature pertaining to anesthesia exposure is imprecise regarding both the duration and timing of exposure. This literature lends itself to the interpretation that exposure durations were brief, or if prolonged, the conceptus may have been aborted and not inspected for signs of teratogenesis. Therefore, it remains unclear whether anesthetic drugs, under alcohol-like exposure conditions, can cause craniofacial and brain structural changes similar to those that alcohol causes in first trimester human fetuses.

#### 6.2.3. Neurobehavioral Disturbances

Mounting evidence from animal research prompted multiple independent research groups to undertake epidemiological studies, the results of which have recently become available. Contrary to expectation, the findings from seven recent studies [96,97,98,99,100,101,102] are in relatively good agreement that school-age children who were exposed to brief anesthesia in early infancy have a significantly increased risk of manifesting neurobehavioral disturbances in psychological domains relevant to both AD/HD and learning disability. Disturbances in both of these domains are understandable, in that the anesthesia-exposed cohorts in most of these studies were exposed to cocktails containing both NMDA antagonist and GABA agonist drugs. While some of the studies [98,99,100] have been interpreted as evidence that it may require multiple exposures or a total exposure duration ≥2 h for a significant learning disability effect, other studies [96,97,101,102] support the interpretation that a single exposure to anesthesia for less than 2 h is sufficient to increase the risk for neurocognitive impairment. 

#### 6.2.4. Tentative Conclusion

The above-cited human findings suggest the unwelcome possibility that currently we may be witnessing a situation with GAs and AEDs that is a replay of the alcohol/FASD story. Over a period of hundreds (or thousands?) of years, alcohol damaged the brains of countless human fetuses, sometimes very severely, and it was not until several decades ago that we became aware that this widely used and abused drug has fetotoxic effects. It is noteworthy that it was gross craniofacial malformations, and not the less conspicuous neurobehavioral disturbances, that first called attention to the fetotoxic effects of alcohol. Studies suggesting that exposure to anesthetic drugs in early pregnancy is not associated with obvious teratogenicity [103] have served to bolster the reputation of GAs as safe drugs for obstetric and pediatric use. This was a reasonable assumption, but is this assumption still tenable in light of new evidence cited above pertaining to exposure of human fetuses or infants to AEDs or GAs, respectively?

## 7. Excessive Cell Death by Apoptosis

### 7.1. Excessive Cell Death during Early Embryogenesis

Numerous studies pertaining to fetal alcohol effects in animals have reported that alcohol exposure during development is associated with loss of cells from several brain regions, especially loss of cerebellar Purkinje cells. In addition, both animal and human studies have reported loss of neuronal mass, or reduced volume of the brain or of specific brain regions. In most studies, methods were not employed that could clarify at a cellular or molecular level the basis for these cellular or volume deficits. In an excellent review article pertaining to Alcohol-induced craniofacial dysmorphism during early gestation, K.K. Sulik [61] pointed out that a common finding in several studies using animal models, is that alcohol exposure during early embryogenesis appears consistently to be associated with excessive cell death. In experiments aimed at clarifying the nature of this cell death phenomenon, Sulik and colleagues [61,62] studied brain changes at serial acute time points following a single exposure to alcohol, and found that excessive acute cell death did occur in specific primordial cell populations that give rise to tissues of the face and brain. Consistent with prior findings by others in alcohol-exposed chick embryos, the mode of cell death was interpreted as apoptosis, and cell death was considered excessive because it was dramatically increased compared to the rate of natural apoptosis (programmed cell death) observed in control brains.

### 7.2. Excessive Cell Death during Later Stages of Gestation

In many recent studies reviewed herein, it has been demonstrated that when alcohol is administered on a single occasion to either rodents or non-human primates during a period equivalent to the human third trimester, there is an acute cell death response in many regions of the brain affecting both neurons and OLs. This cell death response occurs within hours after administration of alcohol and it has been confirmed by a large battery of appropriate methods that the mode of cell death is apoptosis. It has been demonstrated that the cell death response to alcohol is not different in morphological appearance from the apoptotic cell death that occurs naturally in the brain (programmed cell death), but the numbers of cells permanently deleted from various different cell populations by exposure to alcohol is dramatically increased compared to the numbers undergoing natural apoptosis within the same cell populations in control brains. 

### 7.3. Apoptosis—A Unifying Concept

It is well accepted that during normal brain development, there is a balanced and delicately regulated interplay between biological mechanisms that promote cell survival, and mechanisms that promote cell death by apoptosis (cell suicide). For the most favorable biological outcome, a maximum number of properly functioning cells must survive and a maximum number of dysfunctional or redundant cells must be deleted and efficiently removed from the brain. If the balance between cell survival-promoting *versus* cell death-promoting factors is upset by an abnormal circumstance that forces many cells to die that normally would have lived and made a positive contribution to brain function, the outcome will be a deranged and dysfunctional brain, and the nature and degree of derangement will depend on the overall magnitude and timing of the forces that caused the derangement. This analysis does not pretend to clarify mechanisms at a molecular level. It merely draws upon substantial evidence suggesting that alcohol exposure of a developing conceptus at any time from the first to 9th month of gestation can result in relatively acute cell death on a small scale, or on a very large scale, depending on dose, frequency and duration of exposure. There is strong evidence that the mode of cell death is apoptosis, regardless whether alcohol exposure occurs in early embryogenesis when CNS cells are in a proliferative progenitor stage, or in later periods when they have differentiated into neurons and glia and are engaged in the establishment of functional neural networks. 

It is clear from the work of Sulik and colleagues [61] that alcohol exposure early in embyrogenesis can disrupt primordia that are essential for the normal development of the face and brain, and there is no doubt that early disruption of primordial building blocks of the face and brain can cause profound derangements in facial features and in brain structure and function. However, many individuals with FASD do not have obvious craniofacial dysmorphism or gross structural anomalies in the brain, but qualify for the FASD diagnosis based on a wide range of neuropsychiatric disturbances that may be accompanied by relatively subtle structural changes in the brain. Evidence cited herein supports the interpretation that signs and symptoms of FASD, in the absence of obvious craniofacial or brain dysmorphism, can be explained by exposure to alcohol on one or more occasions during later periods of gestation, and that exposure to other apoptogenic drugs (GAs and AEDs) during later periods can also cause FASD-like signs and symptoms. Moreover, regardless whether the offending agent is a GA, AED or alcohol, the best fit explanation for the FASD-like neurobehavioral outcome is that all of these drugs induce apoptotic death and deletion of neurons and OLs (or their precursors) from the developing brain. Thus, we propose that a primary aim of future FASD research should be to develop a better understanding of mechanisms by which alcohol and various apoptogenic pediatric drugs trigger apoptosis of neurons and OLs (or their precursors) at any stage of development. The signaling pathways involved may vary from one gestational stage to the next, but at every stage it is likely to involve pathways that maintain a dynamic equilibrium between factors that promote cell survival and factors that promote cell death by apoptosis. Progress in understanding these mechanisms will hasten the development of methods for preventing FASD-type syndromes, regardless whether they are caused by alcohol or other apoptogenic drugs. 

## 8. Summary and Conclusions

There is compelling evidence that exposure to alcohol during early embryogenesis (4th week of gestation) can cause excessive death of cell populations that are essential for normal development of the face and brain. While this can explain craniofacial malformations and certain structural brain anomalies that sometimes accompany fetal alcohol spectrum disorder (FASD), in many cases these features are absent, and the FASD syndrome manifests primarily as neurobehavioral disorders. Here we have reviewed a growing body of evidence documenting that alcohol triggers widespread apoptotic death of neurons and oligodendroglia (OLs) in the developing brain when administered to animals, including non-human primates, during a period equivalent to the human third trimester of gestation. This cell death reaction is associated with brain changes, including overall or regional reductions in brain mass, and long-term neurobehavioral disturbances. We have also reviewed recent evidence that many drugs used in pediatric and obstetric medicine, including general anesthetics (GAs) and anti-epileptics (AEDs), mimic alcohol in triggering widespread apoptotic death of neurons and OLs in the third trimester-equivalent animal brain, and that human children exposed to GAs or AEDs in late gestation or early infancy have a significantly increased incidence of FASD-like neurobehavioral disturbances. We propose that the mechanism by which alcohol, GAs and AEDs produce neurobehavioral deficit syndromes is by triggering apoptotic death and deletion of neurons and OLs (or their precursors) from the developing brain. We further propose the unifying and research-guiding hypothesis that, although apoptotic cell death is a natural phenomenon during development, pathological augmentation of apoptotic cell death is unnatural, and represents an occult mechanism by which alcohol, or other drugs that mimic alcohol’s apoptogenic action, can disrupt CNS development at any point from early embryogenesis to several years after birth. It follows from this hypothesis that a primary aim of research in this field should be to develop a better understanding of mechanisms by which alcohol and various apoptogenic pediatric drugs trigger apoptosis of neurons and OLs (or their precursors) at any stage of development. Deciphering mechanisms by which these agents trip the apoptosis trigger, will enable the development of methods for preventing that trigger from being tripped, thereby preventing these agents from causing neurodevelopmental disabilities. 
